# *Notes from the Field:* Toxigenic *Vibrio cholerae* O141 in a Traveler to Florida — Nebraska, 2017

**DOI:** 10.15585/mmwr.mm6730a7

**Published:** 2018-08-03

**Authors:** Brianna K.D. Loeck, Amy Roberts, Arryn R. Craney, Stephanie King, Monica S. Im, Thomas J. Safranek, Peter C. Iwen, Anna V. Carlson, Caitlin Pedati

**Affiliations:** ^1^Nebraska Department of Health and Human Services; ^2^Three Rivers Public Health Department, Fremont, Nebraska; ^3^Nebraska Public Health Laboratory, Omaha, Nebraska; ^4^University of Nebraska Medical Center, Omaha, Nebraska; ^5^Division of Global Health Protection and Security, Center for Global Health, CDC; ^6^Oak Ridge Institute for Science and Education, Oakridge, Tennessee; ^7^Division of Foodborne, Waterborne, and Environmental Diseases, National Center for Emerging and Zoonotic Infectious Diseases, CDC; ^8^IHRC, Inc., Atlanta, Georgia.

*Vibrio cholerae* serogroups O1 and O139 are toxigenic strains associated with epidemic cholera; however, other *Vibrio cholerae* serogroups, such as O75 and O141, can also produce cholera toxin, leading to a cholera-like illness identified as vibriosis (*1*). Cholera and vibriosis are more common in the Gulf Coast region of the United States and are related to exposure to coastal water sources and consumption of raw or undercooked shellfish. Persons typically become ill approximately 24–72 hours after exposure. Symptoms can last from 3 to 7 days and range from mild diarrhea to profuse watery diarrhea and vomiting, which can lead to severe dehydration, hospitalization, and death (*2*).

In October 2017, Nebraska state and local health department officials received an electronic laboratory report of stool culture positive for *V. cholerae*. The Three Rivers Health Department in Fremont, Nebraska, began an investigation and completed a patient interview using the Cholera and Other *Vibrio* Illness Surveillance (COVIS)* system form.

The patient, a woman from Nebraska aged 51 years, had recently traveled to Florida. She reported exposure of her feet in the Gulf of Mexico and consumption of raw oysters at a local restaurant. She developed diarrhea approximately 36–48 hours after eating at the restaurant. Upon return to Nebraska, she sought medical care and a stool specimen was collected, from which *V. cholerae* was isolated. The patient was not hospitalized and recovered from the illness. The isolate was forwarded to the Nebraska Public Health Laboratory where additional phenotypic testing presumptively identified the isolate as *V. cholerae.* The isolate was subsequently submitted to CDC for serotyping and molecular testing to confirm the species identification and to test for the presence of cholera toxin genes. The isolate, which was confirmed as *V. cholerae* by *rpoB* gene sequencing, was subsequently identified as serogroup O141 and reported as positive for the *ctxA* cholera toxin gene and the *ompW* gene but negative for the *toxR* and *tcpA* genes. Although genes coding virulence within the *V. cholerae* strains can vary, these virulence genes can be used to assess the epidemic potential of this species. For example, both the *ctx*A gene and the *tox*R gene (the regulatory gene that controls expression of cholera toxin) are commonly found in the O1 and O139 epidemic strains of *V. cholerae*. A traceback investigation by the Florida Department of Agriculture found that the oysters consumed were harvested from the Gulf Coast region in either Florida or Louisiana. No other cases were identified.

Although O141 has not been found to be an outbreak-associated strain, toxigenic *V. cholerae* serogroup O141 has been associated with sporadic illness in the United States (*1*,*3*). These infections have been associated with seafood exposure in Florida and New Jersey and freshwater exposure in Arizona, Michigan, Missouri, and Texas. Since the initiation of COVIS in 1988, 16 cases of toxigenic O141 have been reported in the United States. This is the first case reported from Nebraska.

Vibriosis has been a nationally notifiable condition since 2007. *Vibrio* species are difficult to culture, because they are not easily identified using routine enteric media and often require selective media. However, detection of *Vibrio* species might become more common with increased use of culture-independent diagnostic testing (CIDT) using molecular-based methods, which are considered to be more sensitive than are standard cultures. Some of these CIDT assays are designed to detect a subset of *Vibrio* species and might not identify all cases or only identify cases as vibriosis without species information. For example, the FilmArray Gastrointestinal Panel (BioFire Diagnostics, LLC) has genomic targets for both *Vibrio* species and *Vibrio cholerae.* The manufacturer’s instructions for this assay indicate a limitation for detecting strains of *V. cholerae* that do not carry the *toxR* gene, suggesting that the isolate in this case, without culture follow-up, could have been detected only as a *Vibrio* species. Therefore, CDC asks that state health departments submit all *V. cholerae* isolates for confirmatory testing and additional characterization and suggests that states consider reflex culture testing on specimens identified through CIDT as *Vibrio* species or *V. cholerae*. States that require reporting of vibriosis cases should consider the role confirmatory culture testing plays in the assessment of relevant risk factors to improve public health detection and case management.

## Footnotes

* https://www.cdc.gov/vibrio/surveillance.html.
